# Urban Warming of the Two Most Populated Cities in the Canadian Province of Alberta, and Its Influencing Factors

**DOI:** 10.3390/s22082894

**Published:** 2022-04-09

**Authors:** Ifeanyi R. Ejiagha, M. Razu Ahmed, Ashraf Dewan, Anil Gupta, Elena Rangelova, Quazi K. Hassan

**Affiliations:** 1 Department of Geomatics Engineering, Schulich School of Engineering, University of Calgary, 2500 University Dr. NW, Calgary, AB T2N 1N4, Canada; ifeanyi.ejiagha@ucalgary.ca (I.R.E.); mohammad.ahmed2@ucalgary.ca (M.R.A.); anil.gupta@gov.ab.ca (A.G.); evrangel@ucalgary.ca (E.R.); 2 Spatial Sciences Discipline, School of Earth and Planetary Sciences, Curtin University, Bentley, WA 6102, Australia; a.dewan@curtin.edu.au; 3 Resource Stewardship Division, Alberta Environment and Parks, University Research Park, Calgary, AB T2L 2K8, Canada

**Keywords:** built-up, land surface temperature (LST), local warming, spaceborne remote sensing, surface urban heat island (SUHI)

## Abstract

Continuous urban expansion transforms the natural land cover into impervious surfaces across the world. It increases the city’s thermal intensity that impacts the local climate, thus, warming the urban environment. Surface urban heat island (SUHI) is an indicator of quantifying such local urban warming. In this study, we quantified SUHI for the two most populated cities in Alberta, Canada, i.e., the city of Calgary and the city of Edmonton. We used the moderate resolution imaging spectroradiometer (MODIS) acquired land surface temperature (LST) to estimate the day and nighttime SUHI and its trends during 2001–2020. We also performed a correlation analysis between SUHI and selected seven influencing factors, such as urban expansion, population, precipitation, and four large-scale atmospheric oscillations, i.e., Sea Surface Temperature (SST), Pacific North America (PNA), Pacific Decadal Oscillation (PDO), and Arctic Oscillation (AO). Our results indicated a continuous increase in the annual day and nighttime SUHI values from 2001 to 2020 in both cities, with a higher magnitude found for Calgary. Moreover, the highest value of daytime SUHI was observed in July for both cities. While significant warming trends of SUHI were noticed in the annual daytime for the cities, only Calgary showed it in the annual nighttime. The monthly significant warming trends of SUHI showed an increasing pattern during daytime in June, July, August, and September in Calgary, and March and September in Edmonton. Here, only Calgary showed the nighttime significant warming trends in March, May, and August. Further, our correlation analysis indicated that population and built-up expansion were the main factors that influenced the SUHI in the cities during the study period. Moreover, SST indicated an acceptable relationship with SUHI in Edmonton only, while PDO, PNA, and AO did not show any relation in either of the two cities. We conclude that population, built-up size, and landscape pattern could better explain the variations of the SUHI intensity and trends. These findings may help to develop the adaptation and mitigating strategies in fighting the impact of SUHI and ensure a sustainable city environment.

## 1. Introduction

Urban expansion is one of the fundamental human-induced activities caused by the population growth [1]. It is a fact that the majority of the world population is concentrated in the urban areas [2] that are growing. Natural surfaces are continuously being altered to accommodate the increasing population, which is considered highly in describing the temperature variations in urban areas [3]. The patterns of temperature variations are caused by urban developments and macro climatic fluctuations [4]. Such variations in temperature across cities in the world are associated with several environmental problems [5]. One such problem is the presence of Urban Heat Island (UHI), which is an indication of high temperatures in the urban area compared to the surrounding rural, primarily due to human activities [6,7]. Consequently, it is pertinent to study urban warming in the context of UHI to develop adaptation and mitigation strategies for cities with a sustainable environment. 

Two types of UHI studies were reported in the literature, such as Canopy Urban Heat Island (CUHI) and Surface Urban Heat Island (SUHI), depending on the technique at which the temperature was determined [8]. CUHI is based on in-situ air temperature measurement at the weather stations or through mobile traversing. It is proven to be valuable in defining the air temperature situation [9] in an urban area, but any change in their location or instrument may compromise their usage [10]. Besides, it lacks spatial variability due to its sparse network [11,12]. The spatial variability of station data can be improved using various interpolation techniques of geographic information systems (GIS) [13,14,15,16,17], but it often produces different output maps even with the same input datasets [18,19,20]. In contrast, SUHI depends on land surface temperature (LST) to overcome the issues identified with the measured weather station air temperature [11,21,22,23,24], and it could be acquired through earth observation satellites, i.e., space-borne remote sensing. In addition, satellite remote sensing sensors provide the LST at a low to no cost with different spatial and temporal resolutions [25] as required, and hence, commonly used in the SUHI estimation. For instance, Peng et al. [11] quantified SUHI of 419 global cities and determined their deriving factors using MODIS 8-day composite. The study showed that SUHI driving factors were dependent on the climatic zone of the city. Wu et al. [26] also determine the diurnal, seasonal, and interannual variations of SUHI in South America, and indicated that high daytime SUHI was season-dependent and influenced by land cover. In addition, Imhoff et al. [27] quantified SUHI over 38 most populous cities in the United States, and reported that SUHI increased with the increasing size of the cities, and it was seasonally uneven for the majority number of cities. 

SUHI modulates the local climate [28] as they increase the thermal intensity of urban areas [29], and exposed the urban residence to heatwave, in most cases [30]. Apart from it, SUHI has the potential to impact the local environmental conditions [31], public health [32], energy convection [33], and water consumption [34]. On the other hand, many factors, such as precipitation, aerosol, vegetation, urban form, impervious surface, population, and anthropogenic activities, have been considered to influence SUHI [22,23,35]. Their influence varies from one climatic region to another [36], making adaptation and mitigation strategies mostly to be based on the local climatic conditions [22,37]. Several mitigation strategies, such as planting vegetation, creating parks and green zones, and using construction materials with high solar reflectance and thermal emittance in urban areas, have proven to be very effective in lowering the outdoor ambient temperature, and energy consumption, and carbon emission in many climatic zones. For instance, Wang et al. [38] assessed the possible mitigation of net surface radiation and thermal radiative power using cooling materials on the pavements and roofs and setting urban vegetation. They reported lower than usual heat capacity and temperature of the urban surfaces. Akbari et al. [39] evaluated various materials used to mitigate summertime UHI. The study found three decades of using cool roofs, cool pavements, parks, green zones, green facades, and green walls were effective as mitigation materials to lower the increasing urban temperatures. In addition, large-scale atmospheric oscillations, such as sea surface temperature (SST), Pacific Decadal Oscillation (PDO), Arctic Oscillation (AO), Pacific North America Pattern (PNA), were reported to be impacted the local climate [40] and correlated with local warming [41] in North America. Therefore, these atmospheric oscillations might have some influences on SUHI as well. Moreover, several large water bodies (i.e., Pacific, Atlantic, and Arctic Oceans) surrounding this continent influence atmospheric changes and temperature anomalies. For example, SST anomalies cause atmospheric changes that affect the climate of North America, causing El Niño–Southern Oscillation (ENSO) events. The alternating pressure pattern between western North America and the Pacific Ocean causes PNA that pushes warm temperature toward Canada from autumn to spring [41]. Furthermore, the long-time changing patterns of SST anomalies in the North Pacific Ocean cause PDO that results in long-time heating over the pacific. Lower than average air pressure over the Arctic changes the circulating wind patterns around the North pole and pushes the colder air to the polar regions causing AO [41]. However, we did not find any literature that considered these oscillations as potential influencing factors for SUHI. Further, elevation is considered another influential factor in the estimation of SUHI. High elevated terrains have lower LST values compared to the low-lying areas, even with the similar land cover/land use zone [42], which would likely introduce bias in the SUHI analysis. Therefore, in cities with variable terrain, it is a common practice to remove the LST pixels from remote sensing images corresponding to high and low elevated areas before performing the analysis [43]. However, without removing the unwanted pixels, another approach could be followed as proposed by Hassan et al. [42]. Using the method, LST images could be standardized by applying terrain correction to each pixel with its equivalent standard atmospheric pressure of that pixel’s elevation at a temperature of 20 °C. We did not find any study in the literature that applied this correction method to the LST images for estimating SUHI. 

In Canada, several studies investigated both CUHI and SUHI in some of the major cities [38,44,45,46,47,48,49]. One of the limitations of the CUHI studies was the lack of spatial variability of the temperature data they used and considered the mitigating strategies centered only on meteorological factors and urban forms. For instance, Wang et al. [46] compared the effect of different mitigating strategies in the different urban forms of Toronto city and reported that urban form has a strong mitigating effect on CUHI. Stewart [47] investigated the influence of meteorological conditions on CUHI in Regina (the Capital city of Saskatchewan Province) and reported that CUHI was sensitive to wind conditions and insensitive to humidity and atmospheric pressure. Nkemdirim and Leggat [48] studied the effect of Chinook weather on CUHI in the city of Calgary and showed that it was stronger with poor air quality under chinook weather (a warm wind from the Pacific blows into the lands of the Pacific Northwest in North America) compared to a non-chinook condition. However, limited studies were found in the literature that studied SUHI in Canada, despite having its inherent advantages in characterizing UHI for the cities. For example, a study by Gaur et al. [49] analyzed SUHI in 20 Canadian cities and reported that the magnitude was related to the city’s geographical location, size, and elevation. However, this study failed to access some of the variables, i.e., land cover [27], socio-economic condition [23], and local climate [11] that had a great impact on SUHI. These are very critical to consider as the influencing factors for their effect on the magnitude and trend of SUHI. To overcome all these issues, our overall objective was to quantify SUHI and its trends for The City of Calgary and The City of Edmonton during 2001–2020 using LST data acquired by MODIS at 5.6 km spatial resolution and find the relationships of SUHI with seven potential influencing factors. To fulfill the overall objective, the specific objectives were to (i) apply terrain corrections to the LST images to understand the influence of elevation on LST, (ii) quantify the monthly and annual day and nighttime SUHI, and estimate their patterns and magnitudes of the trends using Mann–Kendall test and Sen’s Slope estimator, (iii) determine the built-up changes in the cities and its relationships with SUHI and population, and (iv) perform the correlation analysis of SUHI with population, precipitation, and indices of SST, PDO, AO, and PNA. We structured this article into different sections, including a description of the study area, data used, mapping of SUHI and its trends, mapping of built-up changes, finding the relationships between SUHI and the influencing factors, results, and discussions.

## 2. Materials and Methods

### 2.1. Description of the Study Area 

**Figure 1:**
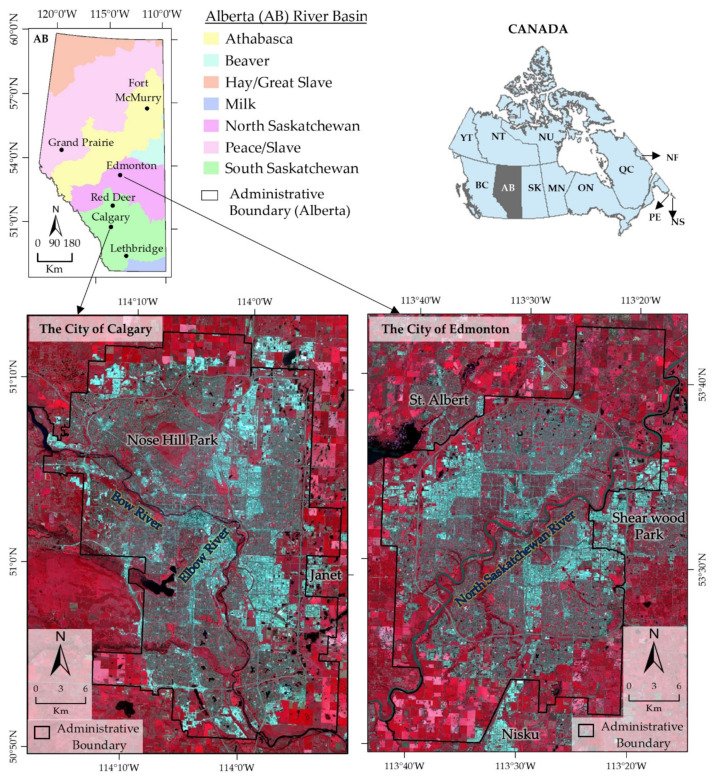
Study area showing the location of the city of Calgary and the city of Edmonton in the Canadian Province of Alberta. The Administrative boundaries of the cities for year 2020 were overlaid on Landsat-8 OLI false color composite images (RGB:5(Near Infrared)4(Red)3(Green)) acquired on 26 July 2020.

We chose the city of Calgary and the city of Edmonton (The Capital) as our study area, the two largest cities in the Province of Alberta, Canada (see Figure 1). The city of Calgary lies between latitude 50°50′ to 51°15′ N and longitude 113°50′ to 114°20′ W with an area of 825.3 km², and the city of Edmonton is situated between latitude 53°20′ to 53°43′ N and longitude 113°15′ to 113°45′ W, covering an area of 684.4 km². Calgary is located within the South Saskatchewan River Basin having the Bow and Elbow rivers traversing through the city from the northwest to the south. The North Saskatchewan River in the North Saskatchewan River Basin traverses through the northeastern part of Edmonton down to the southwest, with many creeks along the river course. In terms of population, Calgary is the most populous city in the province. The population has grown from 878,866 in 2001 to 1,361,852 in 2016 [2] that has reached to 1,047,003 in 2020 (estimated) [50]. Edmonton is the second-most populous city having a population that increased from 666,104 in 2001 to 932,546 in 2016 [2] with an estimation of 1,047,003 in 2020 [51]. Calgary has a daily average minimum temperature of −7.1 °C in January and a maximum of 16.5 °C in July over the period of 1981 to 2010 with total annual precipitation of 418.8 mm [52]. Edmonton has a daily average minimum temperature of −10.4 °C in January and a maximum of 17.7 °C in July, with total annual precipitation of 455.7 mm over the same period [52]. The average elevations of Calgary and Edmonton are 1045 and 645 m above mean sea level with the minimum and maximum of 965 and 1302 m, and 595 and 753 m, respectively. The two cities are cosmopolitan in nature with a landscape that comprises high-rise buildings (concentrated mainly in the city center), recreational parks, green spaces, and some undeveloped lands (agriculture and fallow) located towards the city fringes. Many cities in the province of Alberta witnessed a lot of urban heat challenges in the past years. The most recent was the heatwave of June 2021, resulting in 66 deaths (estimated) [53]. It caused a rise in energy consumption and water demand up to 1.5 times higher than the five-year average. Calgary experienced a historic high temperature of 30 and 36.3 °C for five and two consecutive days, respectively, in the same month [53].

### 2.2. Data Used and Its Preprocessing

We employed nine different datasets in this study that are as follows.

- For mapping SUHI and its trends:
  (i) MODIS monthly LST (MOD11C3 version 6) at 5.6 km from 2001 to 2020 acquired from the National Aeronautics and Space Administration (NASA) that was used for estimating the SUHI;
  (ii) Administrative boundary shapefiles obtained from the geospatial data repository of the city of Calgary and the city of Edmonton were used for the determination of urban and rural areas. We used administrative boundaries of 2001, 2005, 2007, and 2008 for Calgary; and 2001, 2018, and 2019 for Edmonton.
  (iii) Digital Elevation Model (DEM) of Alberta at 25 m acquired from Altalis that was used for standardizing the monthly and annual LST images;
  (iv) MODIS yearly land cover (MCD12Q1 version 6) at 500 m acquired from NASA. It was used for the removal of built-up and water pixels in the rural and urban areas, respectively, from the monthly and annual LST images;
- For mapping the built-up changes:
  (v) Landsat 5 TM and Landsat 8 OLI level 2 products were acquired from the United States Geological Survey (USGS), were used to map the built-up areas in the five-year interval from 2001 to 2020 for the cities. The image dates were 20 June 2001, 5 August 2006, 4 September 2011, 16 August 2016, and 26 July 2020 for Calgary, and 20 June 2001, 30 May 2006, 13 June 2011, 10 May 2016, and 26 July 2020 for Edmonton;
  (vi) For the accuracy assessment of the classified images, we used available very high-resolution satellite images from the Google Earth Pro engine that were having acquisition dates very close to the Landsat images;
- For finding the correlation of SUHI with the influencing factors:
  (vii) A monthly anomaly of the teleconnection indices of the atmospheric oscillations, i.e., Niño 3.4 Sea Surface Temperature (SST), Pacific Decadal Oscillation (PDO), Arctic oscillation (AO), and Pacific North America (PNA), for North America, obtained from NOAA’s National Centers for Environmental Information (NCEI).
  (viii) Monthly average precipitation data of the available stations from Environment Canada; and
  (ix) Population census data for 2001, 2006, 2011, and 2016 from Statistics Canada, and the estimated population of 2020 were derived from the Regional Dashboard of the Government of Alberta.

The acquired MODIS monthly LST product was having 17 layers in HDF format with Geographic (Longitude and Latitude) projection. We preprocessed it to the Universal Transverse Mercator projection system at Zone 12 Northern hemisphere (UTM Zone 12N) with North America Datum 1983 (NAD 83). Next, we prepared the images by extracting the layers with LST values and Quality Control (QC) for day and nighttime (i.e., *LST_Day_CMG* and *QC_day* for daytime, and *LST_Night_CMG* and *QC_Night* for nighttime). Here, the QC layer was used to check for any cloud and aerosol infested pixels that were interpreted by the LST quality assurance with the aid of bit flags. The pixels with an average LST error higher than or equal to 3 K were removed from the images, which were about 0.005% for the daytime and 0.006% for the nighttime LST images in our study. In addition, the acquired surface reflectance images of Landsat 5 TM and Landsat 8 OLI having atmospherically and geometrically corrected by USGS were preprocessed to UTM 12N with NAD 83. 

### 2.3. Methods

**Figure 2:**
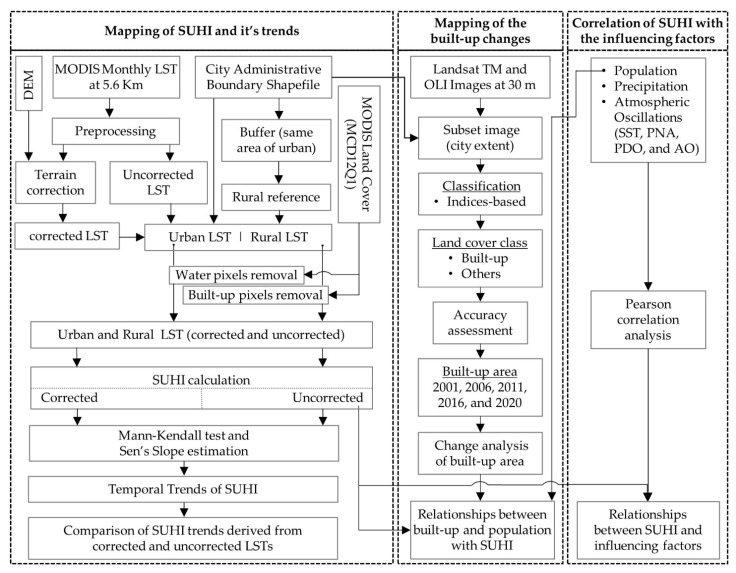
Schematic diagram of the proposed methods followed in this study.

The schematic diagram for the methods we followed is illustrated in Figure 2. It is divided into three stages: (i) mapping of SUHI and its trend, (ii) mapping of built-up changes and its relationship with population and SUHI, and (iii) correlation of SUHI with the influencing factors. A brief discussion of these stages is shown in the following subsections. 

#### 2.3.1. Mapping SUHI and Its Trends

We performed four steps to estimate the SUHI and its trend. In the first step, we applied terrain corrections to the monthly day and nighttime LST images of both cities to derive the standardized LST images. It was performed using Equation (1) by calculating the atmospheric pressure at each LST pixel with its equivalent elevation point in the DEM to standard atmospheric pressure (i.e., 101.3 kPa) at a temperature of 20 °C [42].

(1) $$\theta_{s}=T_{s}\;{\left[\frac{P_{0}}{P}\right]}^{\frac{R}{cp}}$$

where $\theta_{s}$ *=* Terrain corrected surface temperature, $T_{s}$ = Image pixel surface temperature, $P_{0}$ = Average pressure at mean sea level (101.3 kPa), R = Gas constant (287 JKg^−1^ K^−1^), cp = Specific heat capacity of air (1004 JKg^−1^ K^−1^) and P = Atmospheric pressure (kPa) calculated using Equation (2).

(2) $$P=101.3{\left[\frac{293-0.0065Z}{293}\right]}^{5.26}$$

where Z = Elevation in meters (m).

In the second step, we determined the urban and rural areas of the cities for both corrected and uncorrected LST images. For this, we adopted the administrative boundary shapefiles of the cities during 2001–2020 as the urban area. Next, we performed a buffer through a distance outside of the city boundary to define the rural surrounding with approximately the same size as the urban. We opted for this method to avoid the situation where a smaller rural buffer zone or larger urban areas result in a surface urban cool island (SUCI) [54]. Using the city boundary and the rural buffer shapefiles, we prepared the subsets of the LST images (monthly day and nighttime for 2001–2020) for the urban and rural areas, respectively, for the cities. Then, we masked out the water and built-up pixels from the urban and rural areas, respectively, in the LST images. Here, the water and built-up classes (pixels) were derived from the MOD12Q1 data with the International Geosphere-Biosphere Program (IGBP) classification scheme [55]. 

In the third step, we calculated the SUHI of the monthly and annual LST images using Equation (3), both day and nighttime of the cities. First, we estimated the monthly global mean of all the urban and rural LST pixels from 2001 to 2020. Then, we determined SUHI intensity as the difference between the means of the urban and rural areas. We repeated the same processes using the annual images of both cities to determine their SUHI intensity.

(3) $$SUHI=T_{urban}-T_{rural}$$

where *T_urban_* = Mean of urban LST pixels and *T_rural_* = Mean of rural LST pixels.

Finally, we applied Mann–Kendall test [56,57] and Sen’s Slope estimator [58] to the monthly and annual day and nighttime SUHI over the time-series of 2001–2020 to determine the patterns and magnitude of the temporal trends of SUHI. We adopted these statistical methods because they are independent of data distribution and insensitive to data outliers [59]. Note that the annual day and nighttime LST images were prepared by averaging the 12 monthly LST images for each year. To understand the influence of elevation on the day and nighttime SUHI trends, we also performed the linear regressions to calculate the coefficient of determination (R^2^) for both cities between the SUHI trends derived from both corrected and uncorrected LST images.

#### 2.3.2. Mapping of Built-Up Changes 

We performed an indices-based classification on the Landsat images to derive the built-up land cover class in the cities. The class included impervious surfaces such as roads, pavement, parking lots, and residential and industrial buildings. All the remaining land covers were considered as the other class that included vegetation (i.e., shrubs, tree, and grass), open water bodies (i.e., river, lake, and pool), and agricultural lands (cultivated and uncultivated), open spaces, and non-impervious bare surfaces. To implement this classification method, we calculated three indices that included normalized difference vegetation index (NDVI), modified normalized difference water index (MNDWI), and normalized difference built up index (NDBI) from the surface reflectance bands using Equations (4)–(6). These derived index images were then stacked together, and the spectral signatures for the built-up areas were generated and used to derive the land cover map. Note that we applied the indices-based classification method in this study because it performed better than the ISODATA clustering and random forest (RF) classification method in the separation of the built-up land cover of an urban area [25].

(4) $$NDVI=\frac{\rho_{NIR}-\rho_{Red}}{\rho_{NIR}+\rho_{Red}}$$

(5) $$MNDWI=\frac{\rho_{Green}-\rho_{SWIR2}}{\rho_{Green}+\rho_{SWIR2}}$$

(6) $$NDBI=\frac{\rho_{SWIR2}-\rho_{NIR}}{\rho_{SWIR2}+\rho_{NIR}}$$

where $\rho_{NIR}$, $\rho_{Red}$, $\rho_{Green}$, $\rho_{NIR}$, and $\rho_{SWIR2}$ are the surface reflectance for NIR, red, green and SWIR2 spectral bands, respectively.

For the accuracy assessment of the classified thematic images, we prepared a set of reference polygons for the built-up class on the Landsat images with the help of Google Earth Pro images. Next, we calculated a confusion matrix by comparing the thematic classes (built-up, and others) of the classified images with the corresponding classes in the reference polygons for the overall accuracy and Kappa statistics. Further, we calculated the areas of built-up class in the classified images of 2001, 2006, 2011, 2016, and 2020 to determine the changes in the built-up area over the period 2001–2020. Finally, we assessed the relationships of SUHI (calculated in Section 2.3.1) with the changes in the built-up area and census population of the corresponding years by visual representation.

#### 2.3.3. Finding Relationships of SUHI with the Influencing Factors

We performed Pearson’s correlation coefficient (r) analysis between SUHI and each of the potential influencing factors, i.e., population, precipitation, and anomalies of the teleconnection indices (atmospheric oscillations) of SST, PDO, AO, and PNA. To achieve this, we prepared the monthly precipitation data on an annual scale by taking the average of all the station data in a year in the urban area. We also processed the tabular monthly anomalies of the atmospheric oscillations to the annual scale. Finally, we calculated the Pearson correlation coefficients of SUHI for both day and nighttime with precipitation, SST, PDO, AO, and PNA using Equation (7) [60,61], including significance tests at 95 and 99 % confidence levels.

(7) $$r=\left[\frac{\sum_{i=1}^{n}(x_{i}-\bar{x})\;\left(y_{i}-\bar{y}\right)}{\sqrt{\sum_{i=1}^{n}{\left(x_{i}-\bar{x}\right)}^{2}}\;\sqrt{\sum_{i=1}^{n}{\left(y_{i}-\bar{y}\right)}^{2}}}\right]$$

where $x_{i}$ and $y_{i}$ are the x and y variables; $\bar{x}$ and $\bar{y}$ are the mean of the x and y variables; and n the number of observations. 

## 3. Results

### 3.1. Variability in Day and Nighttime SUHI 

**Figure 3:**
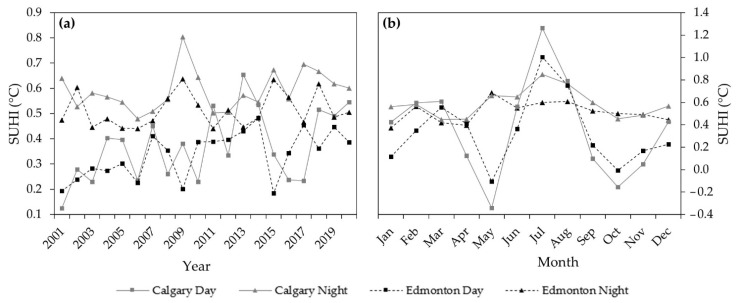
Annual day and nighttime SUHI (**a**); and monthly day and nighttime SUHI (**b**) at the cities of Calgary and Edmonton over the period 2001–2020.

The calculated annual and monthly day and nighttime SUHI based on the observational LST (i.e., uncorrected LST) for Calgary and Edmonton are shown in Figure 3. We observed the highest annual day and nighttime SUHI in 2013 (0.65 °C) and 2009 (0.80 °C), respectively, in Calgary, and in 2014 (0.48 °C) and 2009 (0.64 °C), respectively, in Edmonton (see Figure 3a). On the annual average, we found the highest SUHI during the nighttime (0.59 °C) in Calgary, and the least during the daytime (0.34 °C) in Edmonton (see Figure 3a). The inter annual SUHI variability showed the highest increase in daytime in Calgary with the values of 0.13 to 0.55 °C during the period 2001–2020 compared to Edmonton with values from 0.19 to 0.39 °C over the same period. We also noticed the increase in nighttime SUHI for Calgary from 0.64 °C to 0.68 °C and Edmonton from 0.47 °C to 0.51 °C during the same period. 

The average monthly day and nighttime SUHI of Calgary and Edmonton from 2001 to 2020 are presented in Figure 3b. We found the highest daytime SUHI value of 1.26 and 1.00 °C in July in Calgary and Edmonton, respectively. We also observed the highest nighttime SUHI values of 0.85 °C in July and 0.69 °C in May in Calgary and Edmonton, respectively. We noticed that the negative daytime SUHI occurred in May with the values of −0.34 and −0.11 °C in Calgary and Edmonton, respectively. Our results indicated a considerable variation in the magnitude of day and nighttime SUHI between the cities.

### 3.2. Trends of Day and Nighttime SUHI 

**Figure 4:**
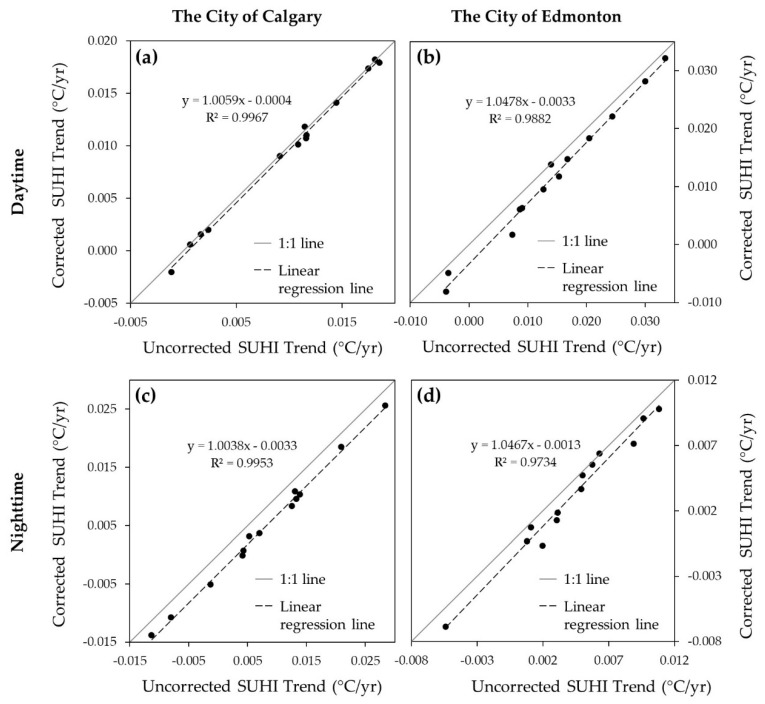
Linear regression between the SUHI trends derived from terrain corrected LST and uncorrected LST during daytime (**a**,**b**) and nighttime (**c**,**d**) for Calgary (**a**,**c**) and Edmonton (**b**,**d**) during 2001–2020.

The comparison between the terrain corrected and uncorrected monthly and annual day and nighttime SUHI trends is presented in Figure 4. We noticed very high values of the coefficient of determination (*R*^2^ > 0.97) during the day and nighttime for both cities. Such a very strong correlation, therefore, indicated that elevation had very little influence on the SUHI trends in the cities, and therefore, we presented a further analysis based on the SUHI derived from the observational (i.e., uncorrected) LST images.

The monthly and annual day and nighttime SUHI trends of Calgary and Edmonton during 2001–2020 are presented in Table 1. We observed a statistically significant increasing trend in the annual daytime SUHI at Calgary (i.e., 0.015 °C/yr) and Edmonton (i.e., 0.012 °C/yr). In the monthly trends of Calgary, we found statistically significant increasing trends in June (0.013 °C/yr), July (with 0.033 °C/yr), August (0.030 °C/yr), and September (0.020 °C/yr) for daytime SUHI; and March (0.028 °C/yr), May (0.021 °C/yr) and August (0.014 °C/yr) for nighttime, respectively. In Edmonton, we found significant increasing daytime SUHI trends in March (0.018 °C/yr) and September (0.009 °C/yr). We also noticed insignificant increasing trends in the nighttime SUHI of Edmonton for all months with the exception of October (−0.005 °C/yr). 

### 3.3. Changes in Built-Up Area

**Figure 5:**
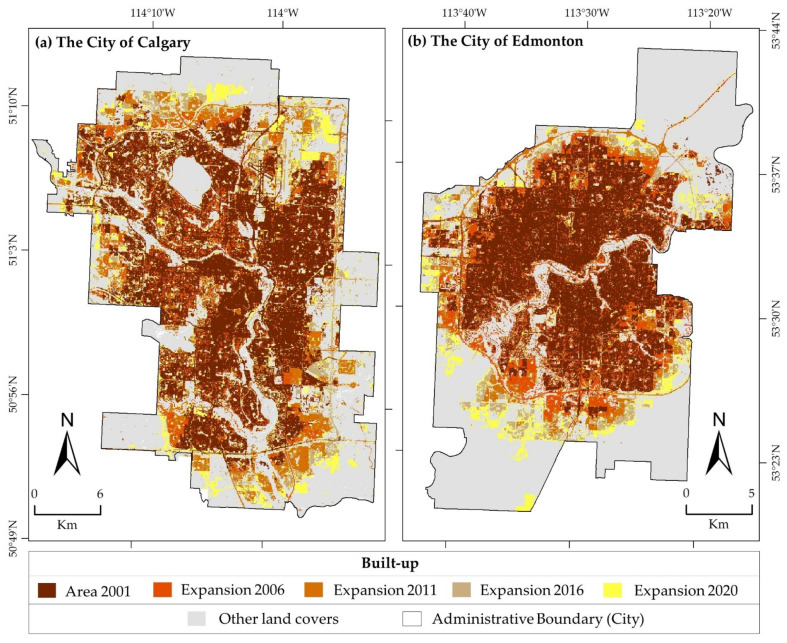
Built-up area changes in the city of Calgary (**a**) and city of Edmonton (**b**) from 2001 to 2020.

The expansions of the built-up area in Calgary and Edmonton from 2001 to 2020 are presented in Figure 5, which were derived from the classified Landsat images. We achieved overall accuracies and kappa coefficients that were greater than 94.76% and 0.91, respectively, for the classified thematic images of 2001, 2006, 2011, 2016, and 2020. The calculated areas of the built-up class in Calgary represented approximately 35.10, 37.52, 42.13, 46.13, and 49.09% of the city in 2001, 2006, 2011, 2016, and 2020, respectively. Whereas areas of the built-up class in Edmonton were approximately 28.56, 32.65, 37.36, 45.81, and 47.38 % of the city in those years, respectively. We noticed an increase in built-up areas in the two cities, about 64.43% in Calgary (from 254.16 to 417.91 Km^2^) and 70.05% in Edmonton (from 200.42 to 340.82 Km^2^), from 2001 to 2020.

### 3.4. Relationships of SUHI with Different Factors 

**Figure 6:**
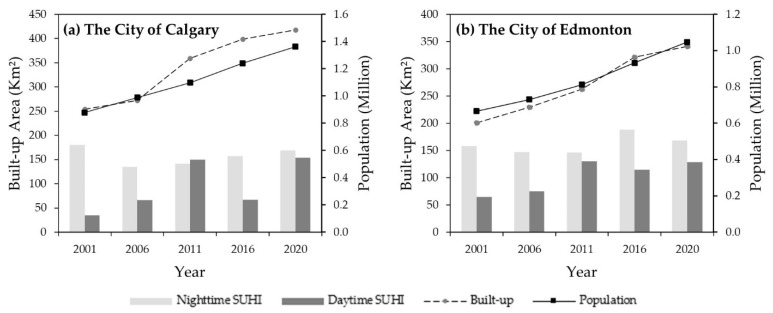
The relationships of SUHI with the built-up area and population for the city of Calgary (**a**) and the city of Edmonton (**b**) in the years 2001, 2006, 2011, 2016, and 2020.

The relationships among the built-up area, population, and annual day and nighttime SUHI over the period 2001–2020 at five-year intervals are presented in Figure 6. We observed that the population of Calgary have increased from 878,866 to 1,361,852 (i.e., 54.96 %) and Edmonton from 666,104 to 1,047,003 (i.e., 57.18 %) over the last 20 years. We also found that SUHI increased at Calgary from 0.13 to 0.55 °C (about 337.08 %) and 0.64 to 0.68 °C (about 6.22 %) for day and night time, respectively, over the study period. We also noticed the increase in SUHI at Edmonton from 0.19 to 0.39 °C (99.58 %) in the daytime and 0.47 to 0.51 °C (6.56 %) in the nighttime. Overall, SUHI increased with the increased population and built-up area over the 20 years with variable proportions.

The correlation analysis of annual day and nighttime SUHI with the potential influencing factors (i.e., population, precipitation, and atmospheric oscillations—SST, PDO, AO, and PNA) at Calgary and Edmonton are shown in Table 2. For the attribution of the relationships, we used a classification scheme of five different categories based on the derived values of Pearson coefficient (i.e., *r*), such as, unsatisfactory (*r* ≤ 0.4), acceptable (*r* = 0.40–0.60), satisfactory (*r* = 0.60–0.70), good (*r* = 0.70–0.85), and very good (*r* = 0.85–1.00) [41]. For instance, we found satisfactory (*r* = 0.67) and very good (*r* = 0.82) positive correlations of the annual daytime SUHI with the population of Calgary and Edmonton, respectively. For the annual nighttime SUHI, we found acceptable (*r* = 0.41) and satisfactory (*r* = 0.61) positive correlation with the population of Calgary and Edmonton, respectively. We also noticed that precipitation has negative but unsatisfactory relationships with the annual daytime SUHI of Calgary (*r* = −0.04) and Edmonton (*r* = −0.15). In contrast, the annual nighttime SUHI showed positive and negative unsatisfactory relationships with the precipitation at Calgary (*r* = 0.06) and Edmonton (*r* = −0.12) respectively. The atmospheric oscillations of SST and PDO indices showed a negative and unsatisfactory relationship with the annual daytime SUHI for the two cities except for an acceptable relationship with SST for Edmonton (*r* = −0.47). In contrast, SST and PDO showed positive but unsatisfactory relationships with the annual nighttime SUHI for the cities, except SST for Edmonton (*r* = 0.46) with an acceptable relationship. In the case of the AO index, we found positive but unsatisfactory relationships with both annual day and nighttime SUHI for the cities apart from a negative relationship (*r* = −0.14) with the annual nighttime SUHI of Calgary. Furthermore, all the PNA correlation coefficients showed positive but unsatisfactory relationships with both annual day and nighttime SUHI for the cities except for a negative relationship (*r* = −0.12) with the daytime at Calgary. 

## 4. Discussion

The strong relationship between the terrain corrected and uncorrected SUHI trends (see Figure 4) we observed was an indication that the elevation factor has little or no influence in the cities. It could probably be due to the minimal elevational difference between the high and low points within the cities. Nevertheless, we found a continuous increase in day and nighttime SUHI over the last 20 years for both cities with a higher magnitude in the city of Calgary. These would probably be related to high population, income, access to social amenities [22], urban form, and surface roughness [9] in the cities. The higher magnitude of SUHI Calgary in compared to Edmonton could probably be related to the city size [27], where the size of Calgary (825.3 km²) is larger than Edmonton (684.4 km²). Large city size has also been reported with a higher magnitude of SUHI compared to small cities in previous studies [22,23,62]. Moreover, a higher annual nighttime SUHI than daytime in both cities was observed. It would probably be explained by the presence of the anthropogenic heat from house heating, lighting, combustion engines, and appliances during the night [11], in addition to daytime heat-trapping [63] caused by impervious surfaces and building’s rooftops [25,39] that released at night. Moreover, the evaporative cooling from urban vegetation during the day could have contributed to the lower daytime SUHI compared with the nighttime [23]. In addition, the highest daytime SUHI in July for both cities could probably be associated with the maximum temperatures recorded in July (summer peak) [25,41] which could potentially increase the daytime SUHI [22]. Conversely, a very weak negative daytime SUHI (i.e., cooling island) in May for both cities would probably be due to the abundance of exposed wet soil in the rural compared to urban. Most of the land in rural is mainly open agricultural land, with some crops in May, having very little or no cooling effect on the temperature. Here, climate change may have caused the early onset of spring [64,65] resulting in the early runoff [66,67] and snowmelt phenomena in the region. It helps the vegetation phenology and wetting of the soil earlier than expected. Wet soil has a high heat-retention capacity and retains more heat than dry soil [68,69], thereby elevating the temperature of the rural more than the urban. Additionally, the evaporative cooling caused by vegetation, rivers, lake, and pond in the urban may have contributed to lowering the urban temperature below that of the rural and caused a weak cooling island.

In the case of the SUHI trends, the observed continuous and significant increase in the annual daytime warming in both cities could probably be due to the continual urban expansion and anthropogenic forcing that resulted from the loss of agricultural land, vegetation, and green space within the urban corridor [70] during the study period. A similar warming pattern was also reported for the same region in other studies [12,41], where Calgary and Edmonton had shown up to 1 and 0.25 °C warming, respectively, during 1961–2010. Additionally, the significant warming trends for daytime SUHI in July and nighttime SUHI in March in Calgary could probably be related to the increasing built-up (thus high proportion of the impervious surfaces) [27], urban structure [46], and land cover composition and configuration [25] in the city. The high proportion of impervious surfaces in the urban, with different spatial arrangements and forms, have higher sensible heat due to their low to no evapotranspirative nature that retains the higher temperature in the urban areas compared to the rural [71]. Besides, the high proportion of impervious land cover, with the densified clustered arrangement, increases the sensible heat aerodynamic resistance as the wind passage within the urban structures is highly reduced, thereby elevating the temperature [25]. In contrast, the non-significant warming trends in Edmonton for all months could be due to the higher proportion of open spaces (especially around the city fringe areas), water, and vegetation within the city boundary that had strong cooling effects on the urban landscape temperature [25]. 

The continuous expansion of the built-up area in the two cities over the last 20 years has been due to an increase in population. It has changed the socioeconomic factors, such as income and occupation that caused the increase in impervious infrastructures such as car parking areas, roads, buildings, etc. These infrastructures were continuously expanded to compensate for the growing population that has encouraged the changes in thermal properties [36], high heat storage, and modification of the energy balance [8] in urban areas. Several studies have also reported a positive influence of population on SUHI [7,22].

In the relationships of SUHI with potential influencing factors, the population showed a strong positive impact on the day and nighttime SUHI of the two cities which might be related to the large built-up size and its composition and configuration. Other studies have also shown similar results that the larger size of urban areas and the compact nature of housing and impervious surfaces were positively related to SUHI [25,27,72]. Whereas precipitation had no noticeable effect on Calgary and Edmonton SUHI because it might be associated with the scale at which precipitation and SUHI were determined [73]. Similar results of having an unsatisfactory correlation of precipitation with SUHI were also reported in other studies [3,73,74]. In addition, the acceptable negative and positive correlations of SST with the annual day and nighttime SUHI, respectively, at Edmonton could probably be due to the effect of El Niño and La Niña events (associated with high and low-temperatures, respectively) on the LST [40] in North America that does not depend on a common seasonal cycle [75]. However, any significant correlation of PNA, PDO, and AO with SUHI in both cities was not observed probably due to the seasonal influence [76,77] on temperature. PNA and PDO have low-frequency variability in the extra-tropical Northern Hemisphere that could only appear in the fall season through early spring in Western Canada, while AO has more influence in the central and eastern parts of Canada only during winter seasons. These findings also adhere to the results reported by Hassan et al. [41] for the same study area.

## 5. Conclusions

In this study, we used MODIS monthly LST to determine SUHI and its trend in the city of Calgary and the city of Edmonton during 2001–2020. We also determined the relationships of SUHI with seven different influencing factors, including the built-up and population in the two cities. We prepared a set of terrain corrected LST images by removing the effect of elevation. Then we determined SUHI as the difference between the mean LST of the urban and rural areas for both uncorrected and corrected sets of images. Next, we estimated the SUHI trends for both datasets and compared their trends. These two datasets showed highly correlated trends, and therefore, we used the observational SUHI trends, i.e., calculated from the uncorrected datasets, in finding the relationships between SUHI and influencing factors such as build-up, population, precipitation, SST, PNA, PDO, and AO. Our analysis revealed that a continuous increase in annual day and nighttime SUHI existed for both cities, where Calgary had the highest magnitude during the nighttime with a significant daytime warming in July. Moreover, SUHI of the two cities was greatly impacted by the increasing population, built-up expansion, and SST over the last 20 years. To combat the increasing SUHI trends and minimize the local warming due to ongoing climate change, more innovative designs in the composition and configuration of urban areas are required for further built-up expansion of the cities. This study would help to comprehend the pattern and drivers of SUHI related to local warming so that the adaptation and mitigation strategies could be developed to achieve sustainable cities and urban environments.

## Figures and Tables

**Table 1:** Monthly and annual day and nighttime SUHI trends (°C/yr) during 2001–2020 in the cities of Calgary and Edmonton. The significant values are in italics, where *, **, and *** indicate the 90, 95, and 99% confidence levels, respectively.

Month	Calgary		Edmonton	
	Daytime	Nighttime	Daytime	Nighttime
January	−0.004	0.004	−0.001	0.010
February	0.009	0.005	0.002	0.001
March	0.024	*0.028* **	*0.018* *	0.003
April	0.007	0.013	0.014	0.011
May	0.014	*0.021* *	0.018	0.005
June	*0.013* *	0.013	0.011	0.009
July	*0.033* **	−0.001	0.011	0.001
August	*0.030* **	*0.014* *	0.019	0.002
September	*0.020* *	0.013	*0.009* *	0.006
October	0.009	0.004	0.002	−0.005
November	0.017	−0.008	0.001	0.006
December	−0.003	−0.011	0.012	0.005
Annual	*0.015* ***	*0.007* **	*0.012* ***	0.003

**Table 2:** Pearson correlation coefficients for annual day and nighttime SUHI against the influencing factors in the cities of Calgary and Edmonton during 2001–2020. The significant values are in italics, where * and ** indicate the 95 and 99% confidence levels, respectively.

SUHI Type	City	Population	Precipitation	Atmospheric Oscillation			
				SST	PDO	AO	PNA
Daytime	Calgary	*0.667* *	−0.035	−0.120	−0.130	0.291	−0.139
	Edmonton	*0.815* **	−0.149	*−0.468* *	−0.140	0.001	0.049
Nighttime	Calgary	0.409	0.059	0.270	0.126	−0.143	0.117
	Edmonton	*0.610* *	−0.118	*0.462* *	0.130	0.003	0.040

## Data Availability

Datasets used in this study are available online: [https://www.ncei.noaa.gov/access/monitoring/products/, (accessed on 11 March 2022)] for the teleconnection indices of atmospheric oscillations of North America, and [https://climate.weather.gc.ca/historical_data/search_historic_data_e.html, (accessed on 11 March 2022)] for the monthly average precipitation data.
